# Experimental demonstration of multiple dimensional coding decoding for image transfer with controllable vortex arrays

**DOI:** 10.1038/s41598-021-91553-0

**Published:** 2021-06-08

**Authors:** Long Zhu, Andong Wang, Mingliang Deng, Bing Lu, Xiaojin Guo

**Affiliations:** School of Communication and Information Engineering, Chongqing University of Posts and Telecommunications, Chongqing, 400065 China

**Keywords:** Optics and photonics, Fibre optics and optical communications

## Abstract

Vortex beams carrying orbital angular momentum (OAM), which featuring helical phase front, have been regarded as an alternative spatial degree of freedom for optical mode coding and multiplexing. For most reported OAM-based mode coding schemes, data information is only encoded by different OAM mode states. In this paper, we introduce a novel design technique to construct vortex array phase grating (VAPGs) for the flexible generation of vortex arrays, and employ the proposed VAPGs to realize multi-dimensional space/mode/amplitude coding/decoding. By designing VAPGs with different parameters and loading them on to a single spatial light modulator (SLM), we successfully generate vortex array with different mode states and relative power in the experiments. Moreover, a 10-bit multi-dimensional space/mode/amplitude data coding/decoding scheme for image transfer in free-space link with a zero bit-error-rate is experimentally demonstrated, which confirm the feasibility of our proposed VAPG-based coding/decoding scheme.

## Introduction

Orbital angular momentum (OAM) beam carrying helical wavefront, also known as vortex beam, has been studied for decades. It was shown by Allen in 1992 that the helically phased vortex beams comprising an azimuthal phase term exp(*ilφ*), possess an OAM of *l*ℏ per photon, where *l* is referred to topological charge and *φ* is azimuthal angle^1^. Due to the unique characteristics, vortex beams have seen wide applications in different areas, such as optical manipulation, optical trapping, optical sensing, and optical imaging^2–8^. Moreover, vortex beams can also be employed for high resolution radar image^9–11^. Theoretically, vortex beams with different mode states are orthogonal with each other. In addition, there are, in principle, infinite OAM mode states, which can be employed as independent information carriers. Hence, these outstanding properties make vortex beams to have been extensively studied to increase the channel capacity for both quantum information communications and classical optical communications^12–14^.

Generally, for vortex beam based optical communications, OAM multiplexing and OAM coding/decoding are two important ways to carry and deliver data information. In OAM multiplexing, multiple collinear vortex beams are used to carry data information, which is known as a subset of mode division multiplexing (MDM) technique. By employing OAM-based MDM, high-capacity data transmission systems are realized in both optical fiber and free-space^15–18^. Diffractive phase gratings are commonly empolyed for OAM mode generation and multiplexing. Recently, digitally programmable metasurfaces are successfully used for OAM-based MDM communications^19,20^. In addition to MDM, the vortex beams carrying OAM can also be regarded as symbols to realize data coding/decoding transmission, which also called OAM shift-keying. In 2004, Gibson demonstrated the transfer of information encoded as OAM modes of a light beam using computer-generated fork phase grating^21^. Since then, lots of works employing vortex beams for data coding/decoding transmission have been demonstrated in free-space, optical fiber and underwater link^22–27^. In theory, the OAM states are theoretically infinite, making it feasible to realize high-dimensional coding/decoding for data transfer in both traditional and quantum communications. Moreover, another key superiority of vortex beam coding/decoding claimed “security”, which does not depend on mathematical or quantum–mechanical encryption methods. However, previous vortex beam coding/decoding schemes mainly concentrate on only mode dimension. To realize *M* bit OAM mode coding/decoding transmission, *2*^*M*^ OAM modes are essential. The number of the employed OAM modes will exponentially increase with the coding bits *M*. Actually, there are still other dimensions of light can be employed for data transmission, such as space and amplitude^28,29^. An efficient way to enhance the information coding/decoding efficiency is the combination of multiple optical dimensions, which can directly improve the communication capacity. By making full use of space, mode and amplitude of light, multi-dimensional data coding/decoding can be achievable.

In this paper, we experimentally demonstrate multi-dimensional space/mode/amplitude data coding/decoding transmission by employing power controllable vortex arrays. We introduce a new approach to design vortex array phase gratings (VAPGs) for the flexible generation of vortex array with mode states and relative power controlled. Different vortex arrays with different OAM states and power distribution are successfully generated in the experiments with high efficiency. In addition, by loading the pre-designed VAPGs on to a single spatial light modulator (SLM), we experimentally demonstrate 10-bit (1024-ary) multi-dimensional space/mode/amplitude data coding/decoding for image transfer in free-space link with a zero bit-error-rate.

## Results

### Concept and principle

**Figure 1:**
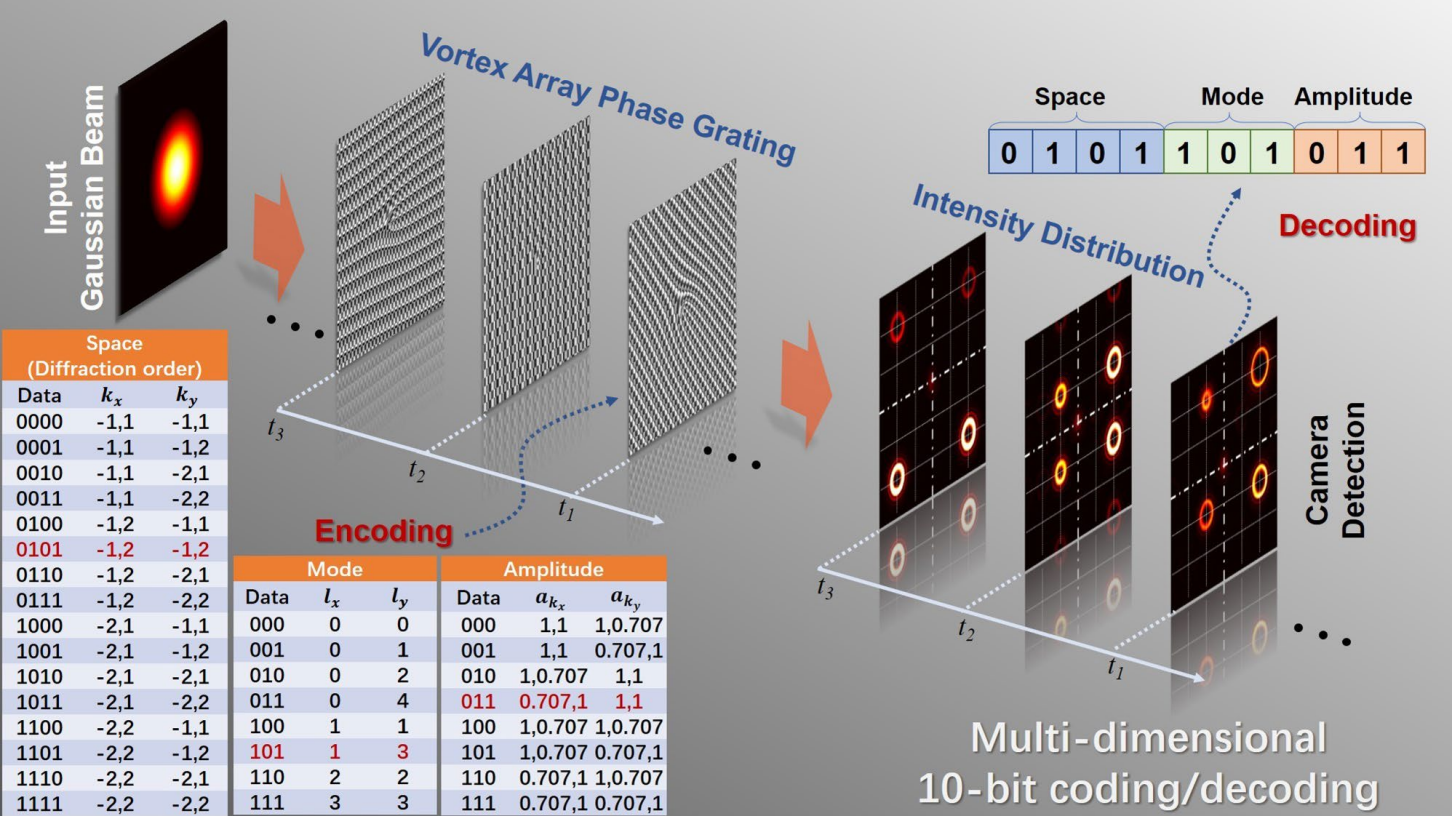
The concept and principle of multi-dimensional space/mode/amplitude coding/decoding using VAPGs. The coding table is shown in the lower-left corner.

The concept and principle of multi-dimensional space/mode/amplitude coding/decoding using VAPGs are shown in Fig. 1. By designing VAPGs with different parameters, one can easily generate vortex array with different OAM mode position, mode states and relative power by a single phase-only element (see “Method” for details). At the transmitter side, the data information can be encoded to the VAPGs generated by setting the corresponding parameters in the coding table (diffraction order, original OAM phase and relative power). When the input Gaussian beam come through the VAPG sequence, vortex array sequence with different intensity distributions can be successfully generated and transmitted. At the receiver side, a camera is employed to record the intensity distribution of the received vortex array. By using simple image processing algorithm, one can easily get the mode position, mode states (diameter of the ring) and the intensity of each mode in the vortex array. Firstly, the recorded intensity images are divided into 4 parts from the center, as shown in Fig. 1. In each part, there is only one OAM mode. The centroiding algorithm is employed to get the position of OAM mode in each part and decode the space data information. Secondly, by calculating the ring diameter of the OAM mode in each part, one can decode the mode data. Finally, by calculating the total intensity of each part, one can get the amplitude information. Thus, the transmitted data can be successfully recovered at the receiver side. In our proof-of-concept experiments, 16 space positions, 8 OAM mode combinations and 8 amplitude combinations are used for multi-dimensional 10-bit data coding/decoding.

### Experimental setup

**Figure 2:**
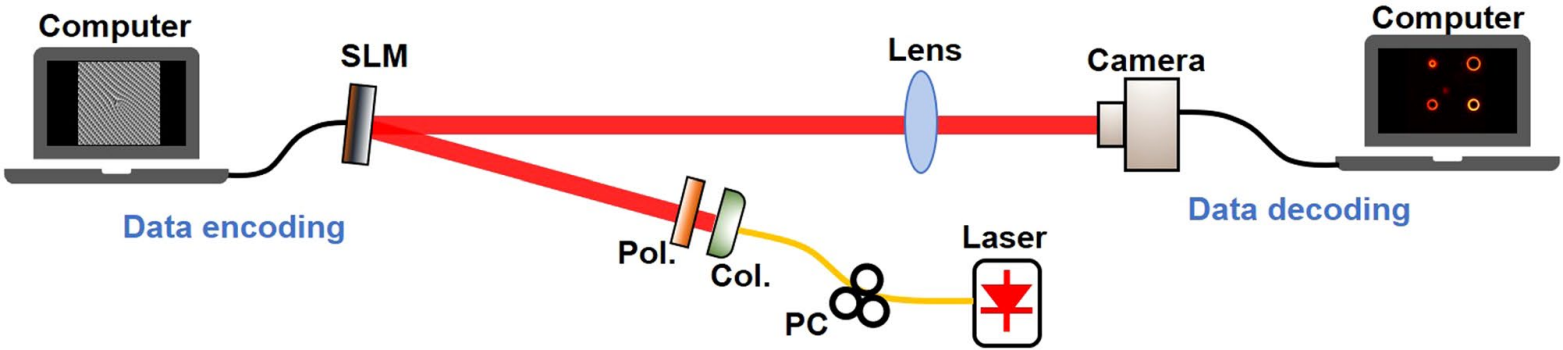
The experimental setup of the high-dimensional space/mode/amplitude coding/decoding data transfer. PC: polarization controller; Col.: collimator; Pol.: polarizer.

The experimental setup of the high-dimensional space/mode/amplitude coding/decoding data transfer is shown in Fig. 2. At the transmitter side, the light comes from the laser with a wavelength of 1550 nm and a linewidth of ~ 1 kHz, and passes through a polarization controller (PC) to adjust its polarization. Then the light is sent to a collimator to generate a Gaussian beam. A polarizer (Pol.) is used to ensure the polarization of the input Gaussian beam along with the working direction of polarization-sensitive SLM (Hamamatsu LCOS-SLM X15223-08). After that, the Gaussian beam come through the SLM, which is loaded with a series of VAPGs. By switching the VAPGs on the SLM controlled by a computer, a coded time-varying vortex array sequence is obtained. At the receiver side, a lens is employed to couple the transmitted vortex array sequence into a InGaAs camera (Hamamatsu C12741-03) for detection. To decode the data information, we divide the recorded intensity image into 4 parts from the center (zero diffraction order), as marked in Fig. 1. Each part has a single OAM mode. By calculating the position, ring width and intensity of the OAM mode in each part, one can easily decode the transmitted data.

### Experimental results of vortex array generation

**Figure 3:**
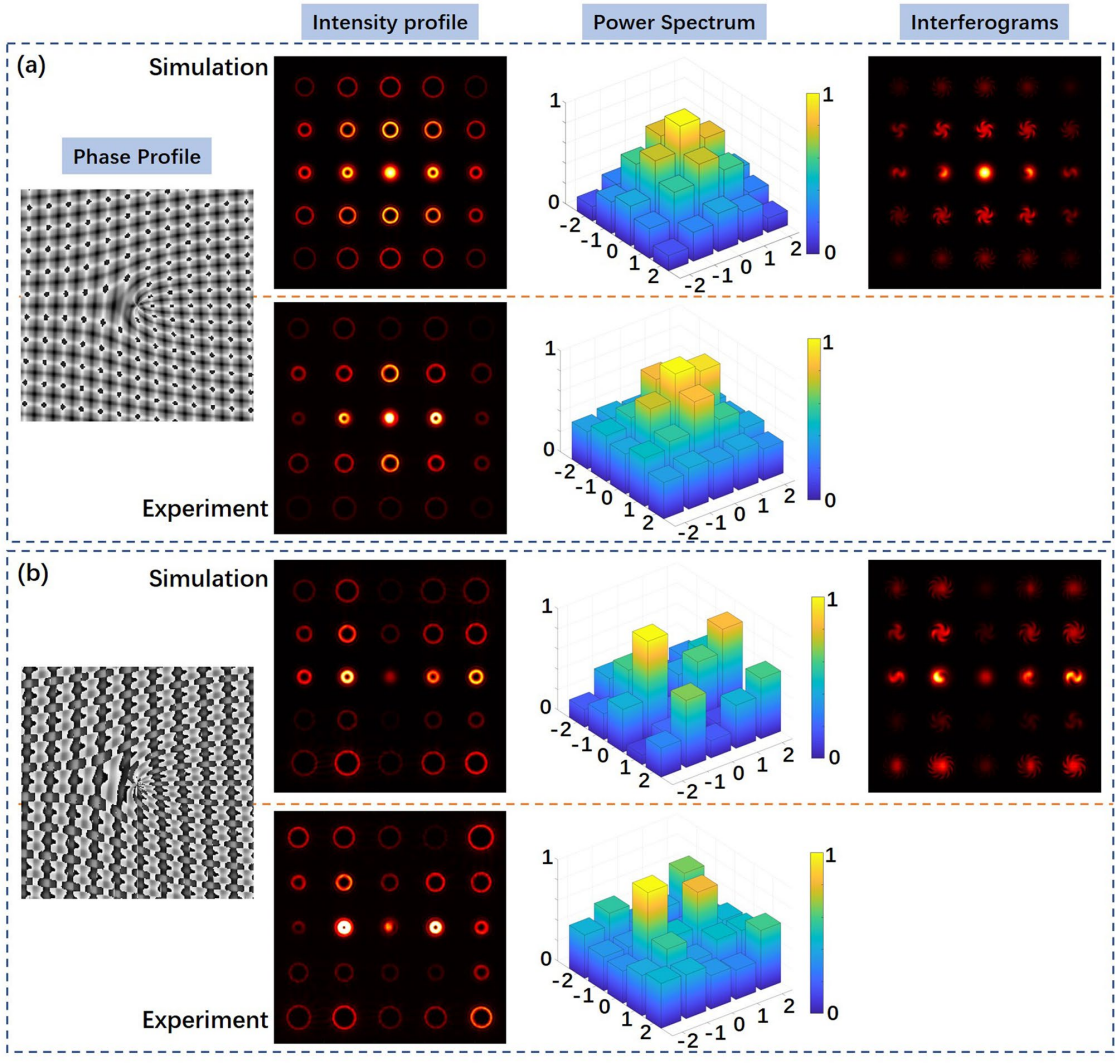
The simulation and experimental results for generating 5 × 5 vortex array. (**a**) The power ratio of the VAPG is set to (0.8:1.5:2:1.5:0.8) in both *x* and *y* direction. (**b**) The power ratio of the grating in *x* direction is set to (2:5:1:3:4), and y direction set to (2:3:5:1:4).

Firstly, we test the generation of vortex array with different power distribution by employing VAPGs in experiments. Figure 3 shows the phase profiles of VAPG, corresponding simulation and experimental results for generation 5 × 5 vortex array (*l* = − 12, − 11, …, 0, … 11, 12) with different power distribution. The simulation results of the light field out come from the VAPG is calculated by Fresnel diffraction integral. The simulated interferograms is depicted to show the mode states of the generated OAM modes in the array. In Fig. 3a, the power ratio of the VAPG is set to (0.8:1.5:2:1.5:0.8) in both *x* and *y* direction. In both simulation and experimental results, one can easily find that the OAM modes at the center is much brighter than the ones at the outside. The calculated power spectrum is also nearly the same, as expected. In addition, the phase distribution of the 2D VAPG in *x* and *y* directions can be set to generate OAM modes with different power ratio. In Fig. 3b, we set the power ratio of the grating in *x* direction to (2:5:1:3:4), and *y* direction to (2:3:5:1:4). The corresponding simulation results shown in Fig. 3b are consistent with the preset power distribution. The captured intensity profile nearly has the same distribution with the simulation one. However, the calculated power spectrum in the experiment has a bit difference with the simulation one, mainly because the limit pixel size and non-ideal phase modulation of the employed SLM. The simulation and experimental results show that by carefully the phase distribution of the 2D VAPG in both directions, one can successfully generate vortex arrays with desired power distribution.

**Figure 4:**
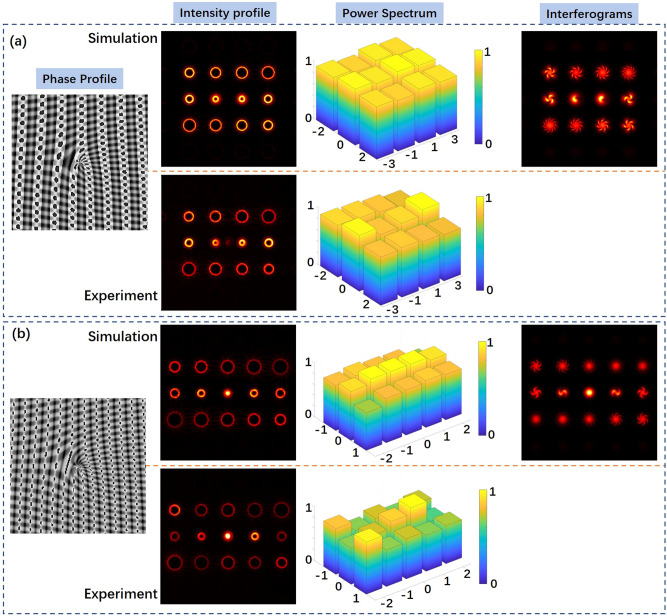
(**a**) The generation of vortex array with odd topological charge from − 11 to 11. (**b**) The generation of vortex array with even topological charge from − 14 to 14.

Moreover, we can also employ gratings with different diffraction orders in *x* and *y* direction to generate some special vortex arrays. Figure 4a shows the generation of OAM modes array with odd topological charge from − 11 to 11. In the phase profile, there are 4 diffraction orders (*k*_*x*_ = − 3, − 1, 1, 3) in *x* direction with spiral phase *l*_*x*_ = 1, and 3 diffraction orders (*k*_*y*_ = − 1, 0, 1) in *y* direction with spiral phase *l*_*y*_ = 8. All the modes in the vortex array are set with the same power. In Fig. 4b, we generate vortex array with even topological charge from − 14 to 14. In the phase profile, there are 5 diffraction orders (*k*_*x*_ = − 2, − 1, 0, 1, 2) in *x* direction with spiral phase *l*_*x*_ = 2, and 3 diffraction orders (*k*_*y*_ = − 1, 0, 1) in *y* direction with spiral phase *l*_*y*_ = 10. One can clearly see the topological charge and the relative power of generated OAM modes in the simulated intensity profile and the interferograms. The simulation and experimental results indicate that one can successfully generate vortex arrays with different mode states distributions by carefully designing the VAPGs.

### Experimental results of high-dimensional space/mode/amplitude coding/decoding

**Figure 5:**
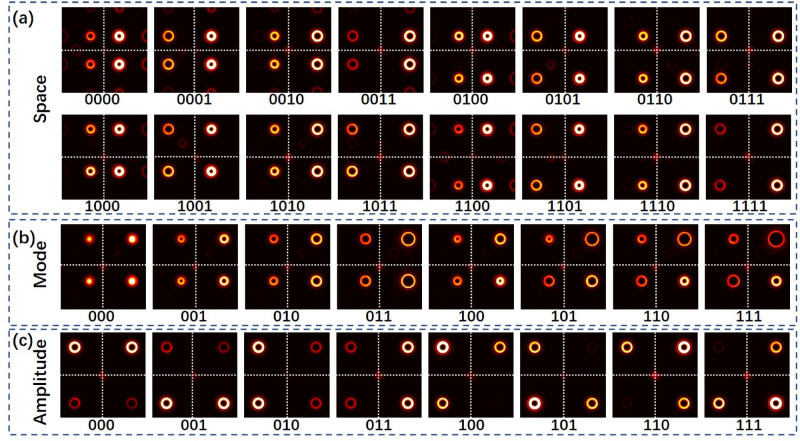
Examples of recorded vortex array intensity patterns for space/mode/amplitude coding/decoding. (**a**) 16 space coding intensity patterns with different diffraction orders; (**b**) 8 recorded intensity patterns with different mode states; (**c**) 8 recorded intensity patterns with different amplitude distribution.

By employing VAPGs, we successfully generate vortex array with different mode distribution and relative power. Here, by switching the prepared VAPGs on the SLM, we can realize multi-dimensional data coding/decoding in experiments. Figure 5 shows some examples of recorded vortex array intensity patterns for space/mode/amplitude coding/decoding. The cross dashed line marked at the center of the intensity image is used to make the position of the OAM modes clear to identify. The parameters for the generation of the vortex arrays are selected according to the coding table shown in Fig. 1. In Fig. 5a, we show 16 space coding intensity patterns by choosing different diffraction orders in *x* and *y* direction. The mode and amplitude parameters of the VAPGs are all set to *l*_*x*_ = 0, *l*_*y*_ = 2 and ${a}_{{k}_{x}}$ = (0.707, 1), ${a}_{{k}_{y}}$ = (1, 1). From the received intensity patterns, one can easily distinguish the position of OAM modes in each patten and decode the data information. Figure 5b shows the 8 recorded intensity patterns with different mode states for data coding. The space and amplitude parameters of the VAPGs are all set to *k*_*x*_ = (− 1, 2), *k*_*y*_ = (− 1, 2) and ${a}_{{k}_{x}}$ = (0.707, 1), ${a}_{{k}_{y}}$ = (1, 1). As the ring diameter of OAM mode is determined by the mode states (topological charge), one can get the ring diameter of each OAM mode in the vortex array by using simple image processing algorithm, and decode the data information. At last, we show the recorded intensity patterns with different amplitude distribution in Fig. 5c. The space and mode parameters of the VAPGs are all set to *k*_*x*_ = (− 2,2), *k*_*y*_ = (− 2,2) and *l*_*x*_ = 0, *l*_*y*_ = 2. Seen from Fig. 5c, the amplitude distribution of each recorded pattern can be easily recognized. Thus, the data encoded vortex array can be successfully decoded from the received intensity patterns by the camera.

**Figure 6:**
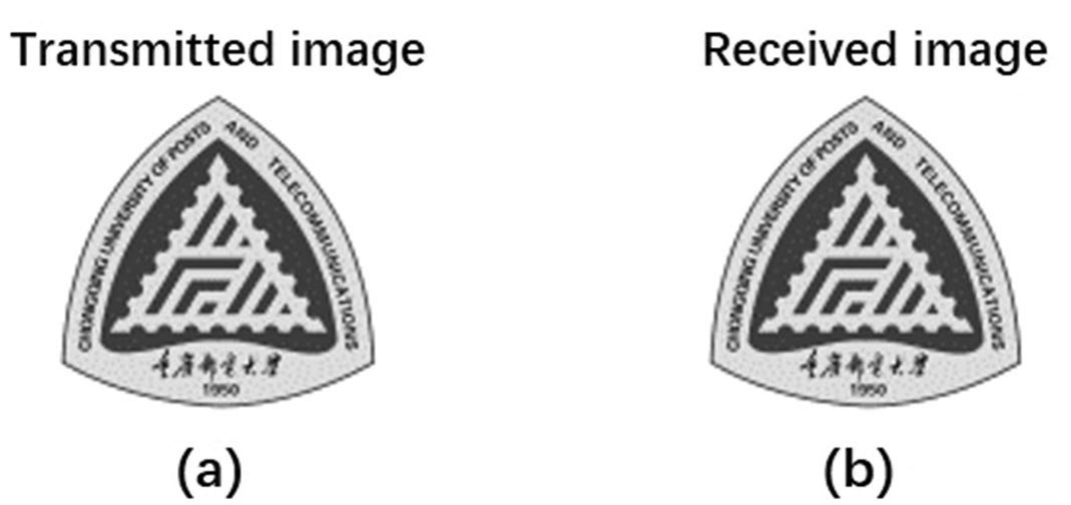
The transmitted and received grayscale images using the proposed multi-dimensional space/mode/amplitude data coding/decoding scheme.

In addition, to vividly demonstrate the data transmission performance of the proposed high-dimensional space/mode/amplitude data coding/decoding, an 80 × 80 pixels image with 256 Gray-scale levels is transmitted in free-space as shown in Fig. 6a. Each pixel of the gray image carries information of 8 bits, implying such image is 51.2 Kbit in total. Hence, the 80 × 80 pixels gray-scale image can be converted to 5120 10-bit symbols, which can be mapped to 5120 VAPG pattern sequence. By switching the corresponding VAPGs on the SLM, the image is coded and transformed into a series of time-varying vortex arrays. After free-space transmission, the vortex arrays are detected by the Camera for decoding. Figure 6b shows the received image recovered by the receiver, which exactly recovers the transmitted one with zero bit-error-rate. The obtained results indicate successful implementation of high-dimensional space/mode/amplitude data coding/decoding with favorable transmission performance.

## Discussion

In summary, we have presented a new approach to design VAPGs for the flexible generation of vortex array with high efficiency. Different vortex beams with different desired power distribution are successfully generated in the experiments. Moreover, by loading the pre-designed VAPGs on to a single SLM, we experimentally demonstrate 10-bit (1024-ary) high-dimensional space/mode/amplitude data coding/decoding for image transfer in free-space. An 80 × 80 pixels gray-scale image is successfully transmitted with zero bit-error-rate. In our proof-of-concept experiments, 16 space positions, 8 OAM mode combinations and 8 amplitude combinations are used for high-dimensional 10-bit data coding/decoding. In the future, more space positions, OAM modes and amplitude levels can be employed to further improve the data rate by employing high precision optical devices, such as metasurfaces. In addition, the flexible approach to generate vortex array may open a door to facilitate more applications in optical manipulation and optical communications using OAM modes.

## Method

**Figure 7:**
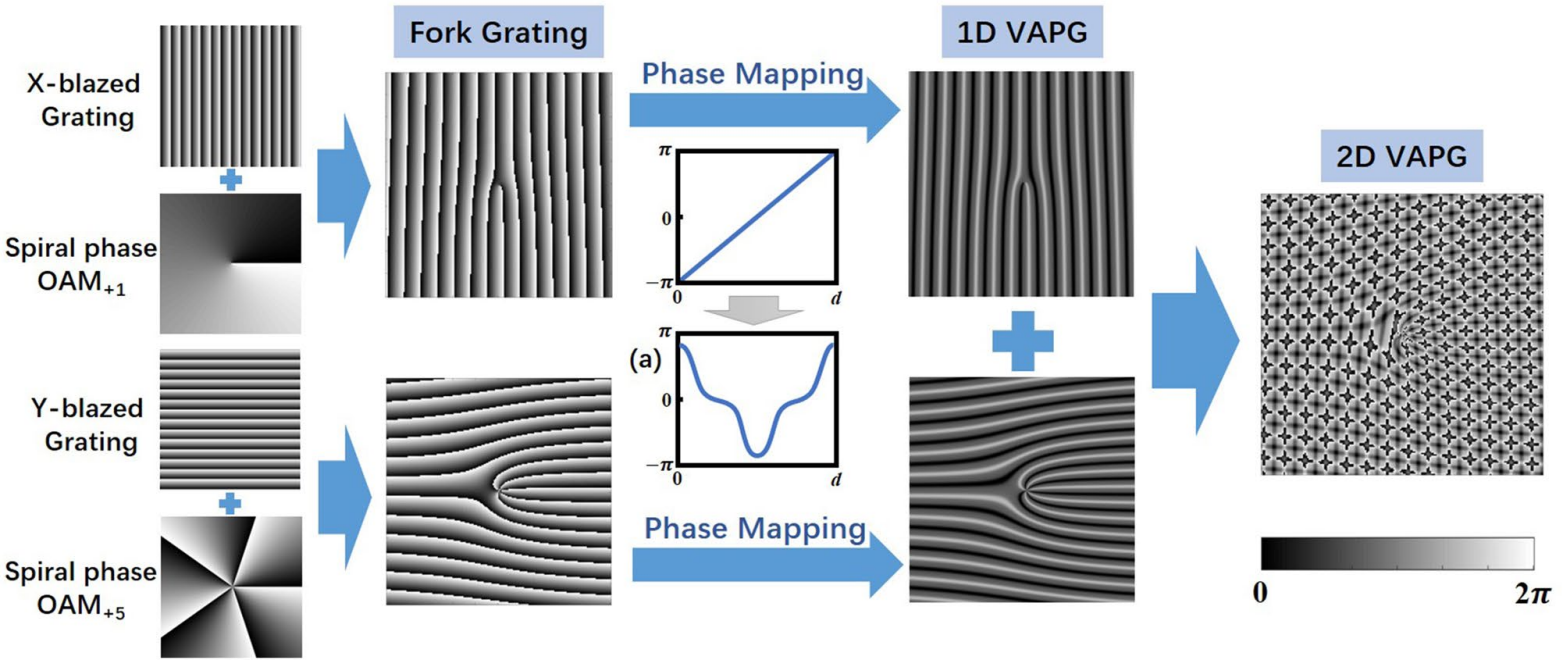
The construction process of 2D VAPG for flexible generation of vortex array.

In this section, we introduce the construction process of 2D VAPG for flexible generation of vortex array, as shown in Fig. 7. Firstly, we get two fork gratings for generating single OAM mode (OAM_+1_ and OAM_+5_ in Fig. 7) in *x* and *y* direction. The fork gratings are generated with blazed grating and spiral phase distribution of OAM mode, which can only generate one OAM mode at the first diffraction order. In order to control the power of the OAM modes at each different diffraction order flexibly, one need to carefully design the phase profile of the grating.

Here, we propose an iterative algorithm to optimize the phase distribution of the grating. The phase grating can be characterized by a periodic function $\phi (x) = \phi (x + 2\pi )$ that changes the phase of an input beam at *x* direction by the amount $\phi (x)$. A uniform light beam passing through this grating will get split up into a large number of beams according to diffraction theory. The amplitude of these beams in different diffraction order are determined by the Fourier coefficients of $e^{i\phi (x)}$. The amplitude of the different diffraction order $k$ can be expressed as

1 $$ a_{k} = \frac{1}{2\pi }\int_{ - \pi }^{\pi } {e^{i\phi (x)} e^{ - ikx} dx} ,\;\;k = 0, \pm 1, \pm 2 \ldots $$

which is only determined by periodic phase $\phi (x)$ of the grating. Thus, by controlling the periodic phase $\phi (x)$, one can directly control the relative output power of each diffraction order. The designing of the periodic phase distribution $\phi (x)$ is a minimization problem. Here, we introduce a method to construct desired phase. The phase profile can be defined as $\phi (x) = exp[if(x)]$ with the phase function

2 $$ f(x) = {\text{Re}} \left\{ { - i\ln \left[ {\sum\limits_{k = - \infty }^{\infty } {B_{k} \exp (ikx)} } \right]} \right\}. $$

In Eq. (2), Re{} means “real part of”, and $B_{k}$ is a decisive factor for $\phi (x)$. Discarding the imaginary part of the right-hand side of Eq. (2) is equivalent to setting amplitude to unity, which ensures $\phi (x)$ is a phase-only function. Then, we can expand the construct phase $\phi (x)$ in Fourier series:

3 $$ \phi (x) = \sum\limits_{k = - \infty }^{\infty } {C_{k} } \exp (ikx). $$

In order to achieve high efficiency, the decomposition coefficient $C_{k}$ should have little difference with $a_{k}$. Here, we introduce a parameter of relative root-mean-square error (R-RMSE) to evaluate the difference:

4 $$ R{\text{-}}RMSE = \sqrt {\frac{{\sum\nolimits_{m = 1}^{n} {\left( {\left| {C_{{l_{m} }} } \right|^{2} - \left| {A_{{l_{m} }} } \right|^{2} } \right)^{2} } }}{{n\sum\nolimits_{m = 1}^{n} {\left| {C_{{l_{m} }} } \right|^{2} } }}} . $$

The smaller of the R-RMSE, the better performance of the phase-only element we can achieve. Since the desired weight coefficients $a_{k}$ is settled at first, the parameter R-RMSE is determined by $C_{k}$ or $B_{k}$. Then it becomes a simple minimization problem. We need to find the suitable $B_{k}$ for minimizing R-RMSE. To solve the problem, we have proposed pattern search assisted iterative (PSI) algorithm^30^. It is a highly effective method for generating multiple collinear OAM modes with a single phase-only element. In this work, the PSI algorithm is applied to design phase gratings for vortex beam array generation.

**Figure 8:**
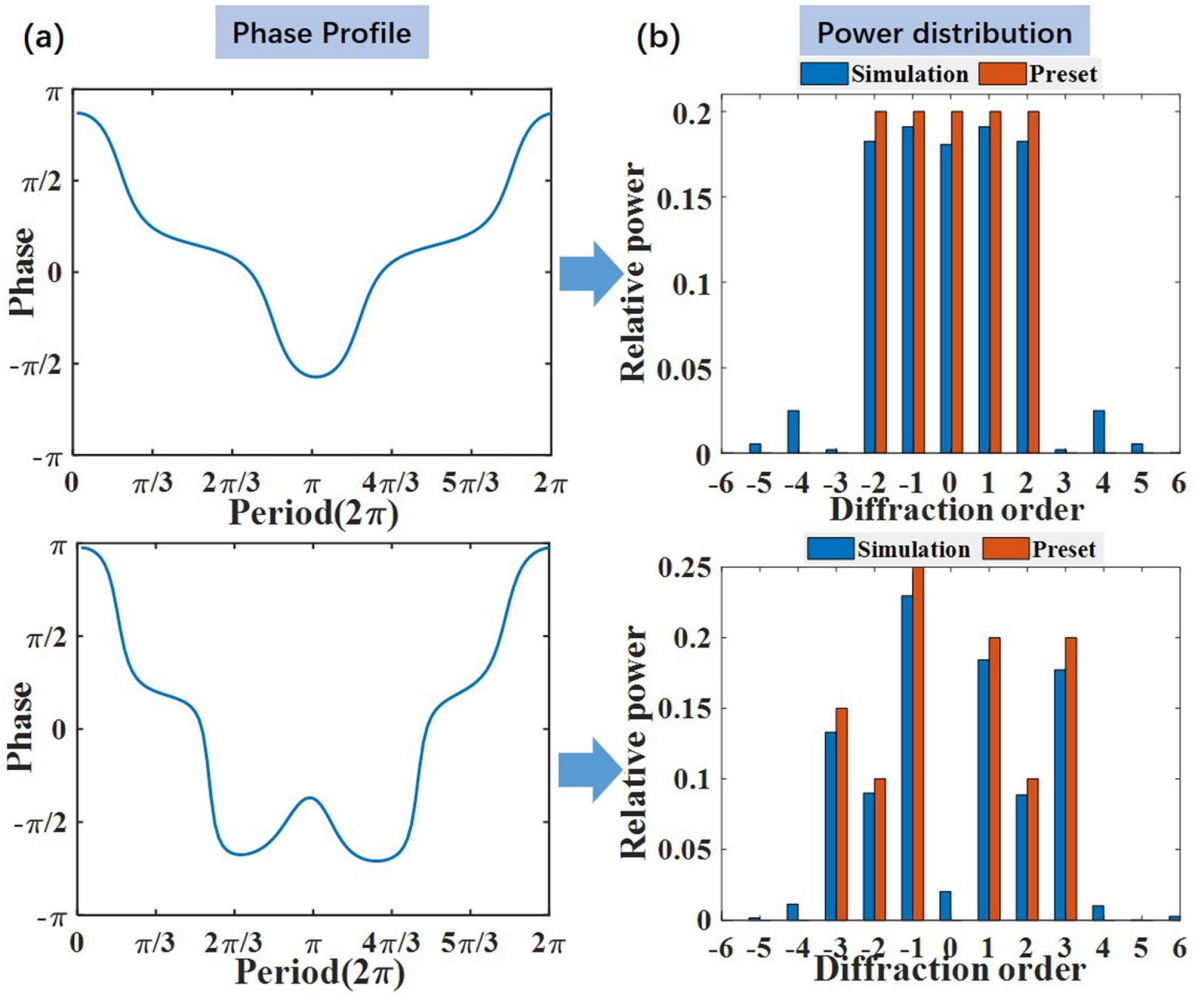
Phase gratings for generating OAM modes with power controlled. (**a**) Phase profiles, (**b**) calculated power distribution of each diffraction order.

By using the method described above, we can arbitrarily manipulate the diffraction order and relative power of grating. Figure 8 shows two generated phase profiles of grating in one period and the corresponding output power distribution. The first grating generates 5 diffraction orders with equal power. The second one generates 7 diffraction orders with different relative power. Seen from Fig. 8, one can find that the generated phase grating can successfully control the diffraction order and relative power. The simulated results are nearly the same with the preset output power distribution. In addition, the calculated diffraction efficiencies of the two gratings are 92.8% and 90.3%, which shows favorable performance.

After that, the linear phase profile in fork grating pattern is mapped to the corresponding desired phase profile (Fig. 7a) generated by PSI algorithm. Then, we can get two complex phase profiles, which can generate 1D vortex array which desired relative power. At last, we add two phase profiles in *x* and *y* direction to generate 2D phase grating for vortex array generation as depicted in Fig. 7. The topologic charge of the OAM modes in the vortex array is determined by the origin spiral phase l and the diffraction order *k*. In 1D VAPG, the topological charge of the target OAM mode in the array is *l* × *k*. In 2D VAPG, the topological charge of OAM modes at each diffraction order in the array is *l*_*x*_*k*_*x*_ + *l*_*y*_*k*_*y*_, where *l*_*x*_ and *l*_*y*_ are the origin spiral phase of the x and y direction fork grating, *k*_*x*_ and *k*_*y*_ are the diffraction order of the target OAM mode in x and y direction, respectively. The relative power of each diffraction order in the vortex array can be expressed as ${\left({a}_{{k}_{x}}{a}_{{k}_{x}}\right)}^{2}$. Thus, we can generate vortex arrays with controlled OAM mode states and relative power by setting the corresponding parameters (diffraction order *k*_*x*_ and *k*_*y*_, original spiral phase of OAM mode *l*_*x*_ and *l*_*y*_, and relative power ${a}_{{k}_{x}}$ and ${a}_{{k}_{y}}$) of the grating in x and y directions.

## Data Availability

The datasets generated or analysed during the current study are available from the corresponding author on reasonable request.
